# Supplementary material for: Roles of Physicochemical and Structural Properties of RNA-Binding Proteins in Predicting the Activities of Trans-Acting Splicing Factors with Machine Learning
Source: Int J Mol Sci. 2022 Apr 17;23(8):4426. doi: 10.3390/ijms23084426 (PMC9030803; doi:10.3390/ijms23084426)
Supplement: Supplementary file 1 [file ijms-23-04426-s001.zip › ijms-1645581-supplementary.pdf]

Roles of Physicochemical and Structural Properties of RNA Binding Proteins in Predicting the Activities of Trans-Acting Splicing Factors with Machine Learning

***Lin Zhu*[1] *and Wenjin Li*[1,*]**

[1] *Institute for Advanced Study, Shenzhen University, Shenzhen 518060, China*

Correspondence:

Wenjin Li, Room 720, Institute for Advanced Study, Shenzhen University, Shenzhen 518060, China;

E-mail: liwenjin@szu.edu.cn; Tel: +86-0755-26942336

Supplementary materials

Table S1: The performance of 647 features with the number of component ranging from 1 to 10.

Table S2: mRMR features list.

Table S3: The Spearman's correlation coefficient of the mRMR features list.

Table S4: Forward feature list.

Table S5: Spearman's correlation coefficient of the forward features list.

Table S6: Performance of Wang's features with the number of components ranging from 1 to 10.

Table S7: Original dataset consists of 85 splicing factors.

Table S8: Conversion of 85 experimentally tested splicing factors into a 700-dimensional feature matrix by feature encoding.

Table S9: Construction of a 647-dimensional feature matrix from the 700-dimension feature matrix by removing the features that are almost 0 in all samples.

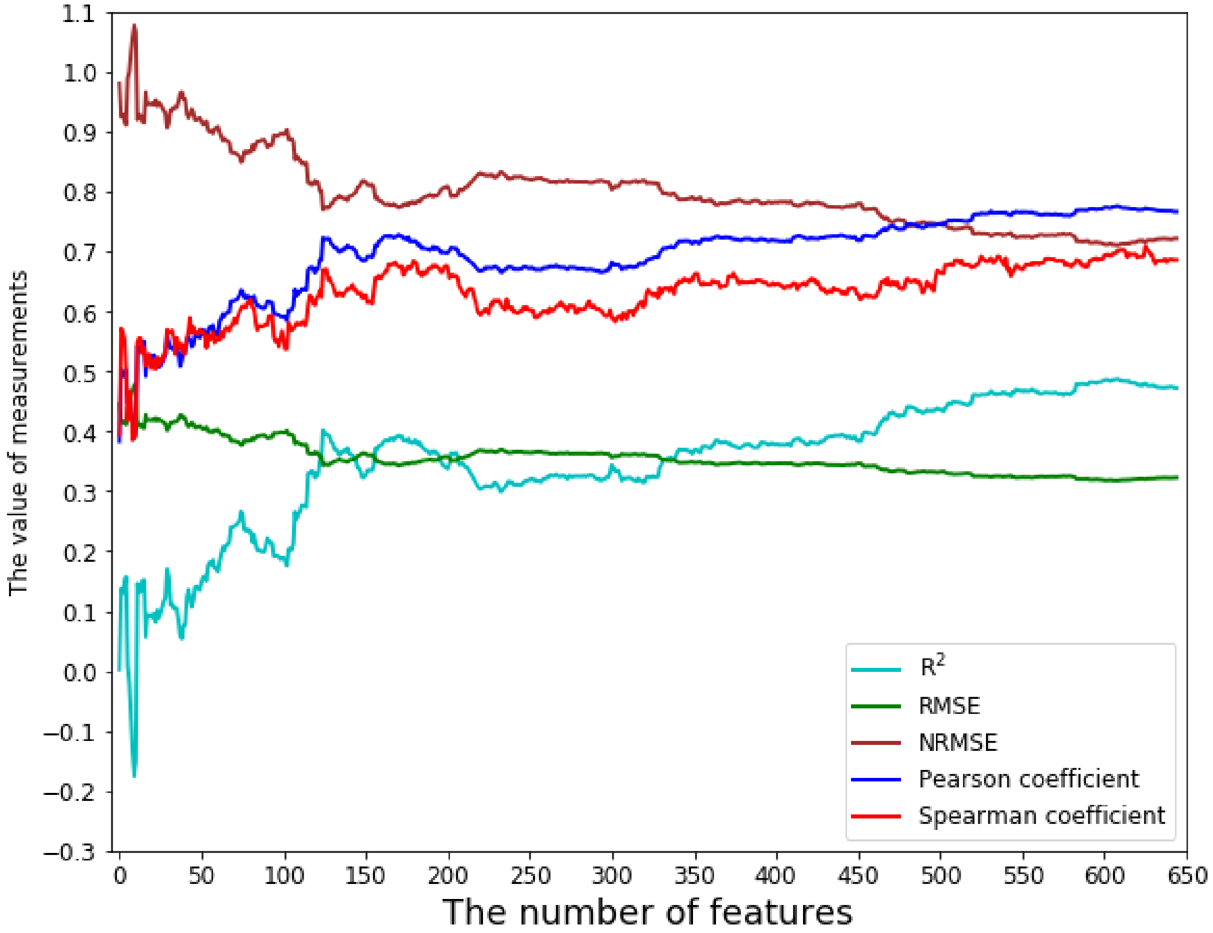

Figure S1: Curves of five metrics produced by the mRMR features. Three metrics R^2, Pearson's coefficient and Spearman's coefficient show a similar uptrend, and $RMSE$ and $NRMSE$ display a similar downtrend.

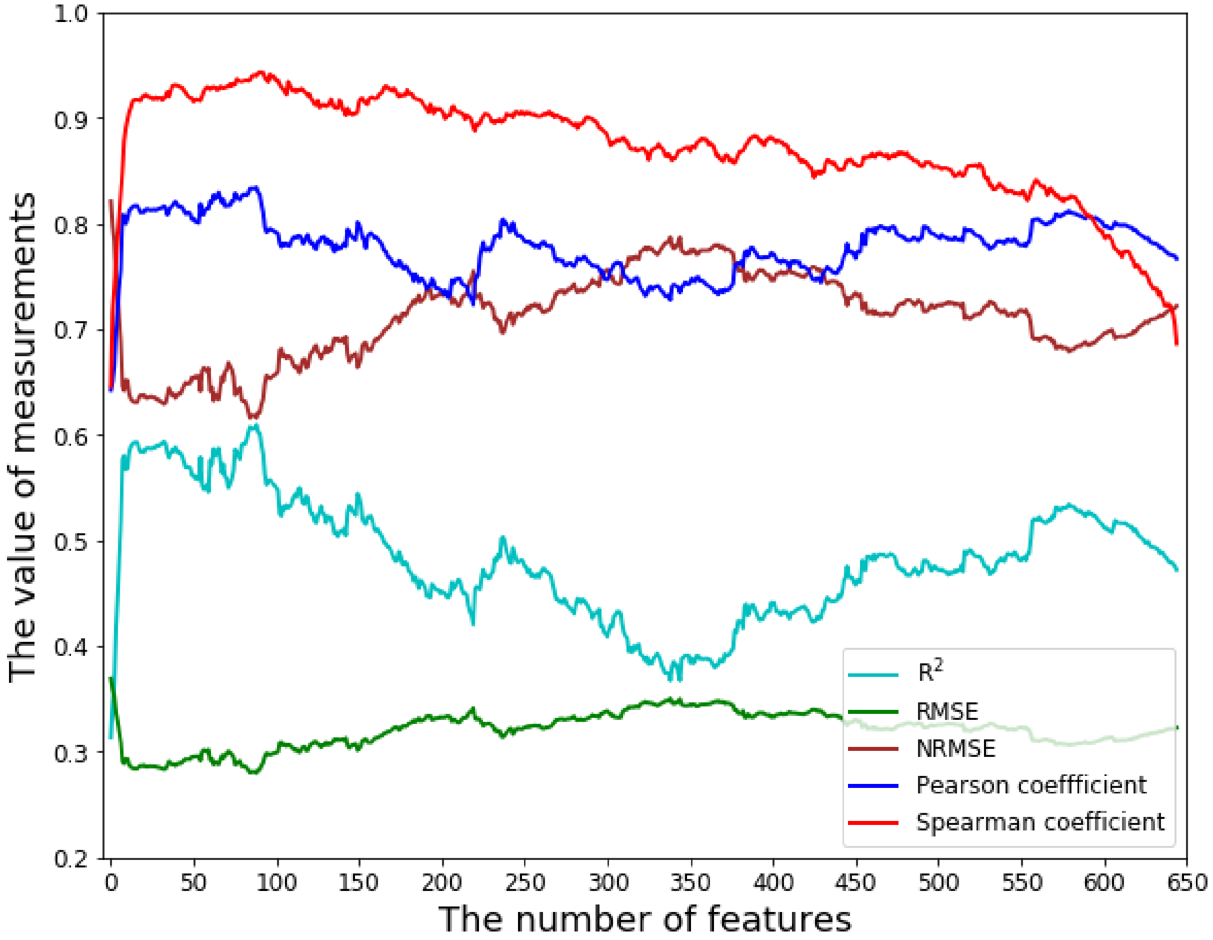

Figure S2: Curves of five metrics produced by using the forward feature searching strategy. Three metrics R^2, Pearson's coefficient and Spearman's coefficient show a similar trend, and $RMSE$ and $NRMSE$ display a similar trend.

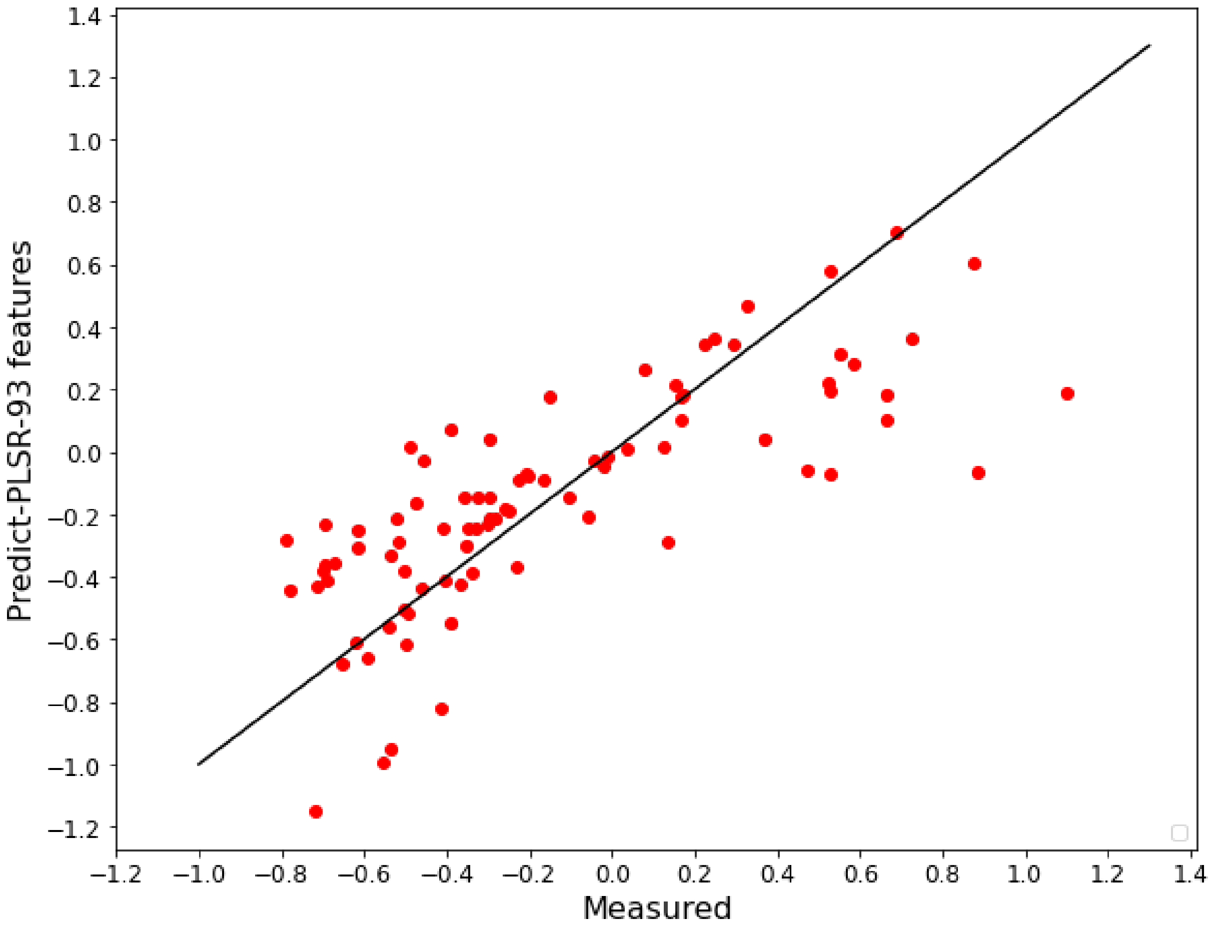

Figure S3: Fitting graph produced by 93 features, showing the best feature subset (93 features) with good performance.

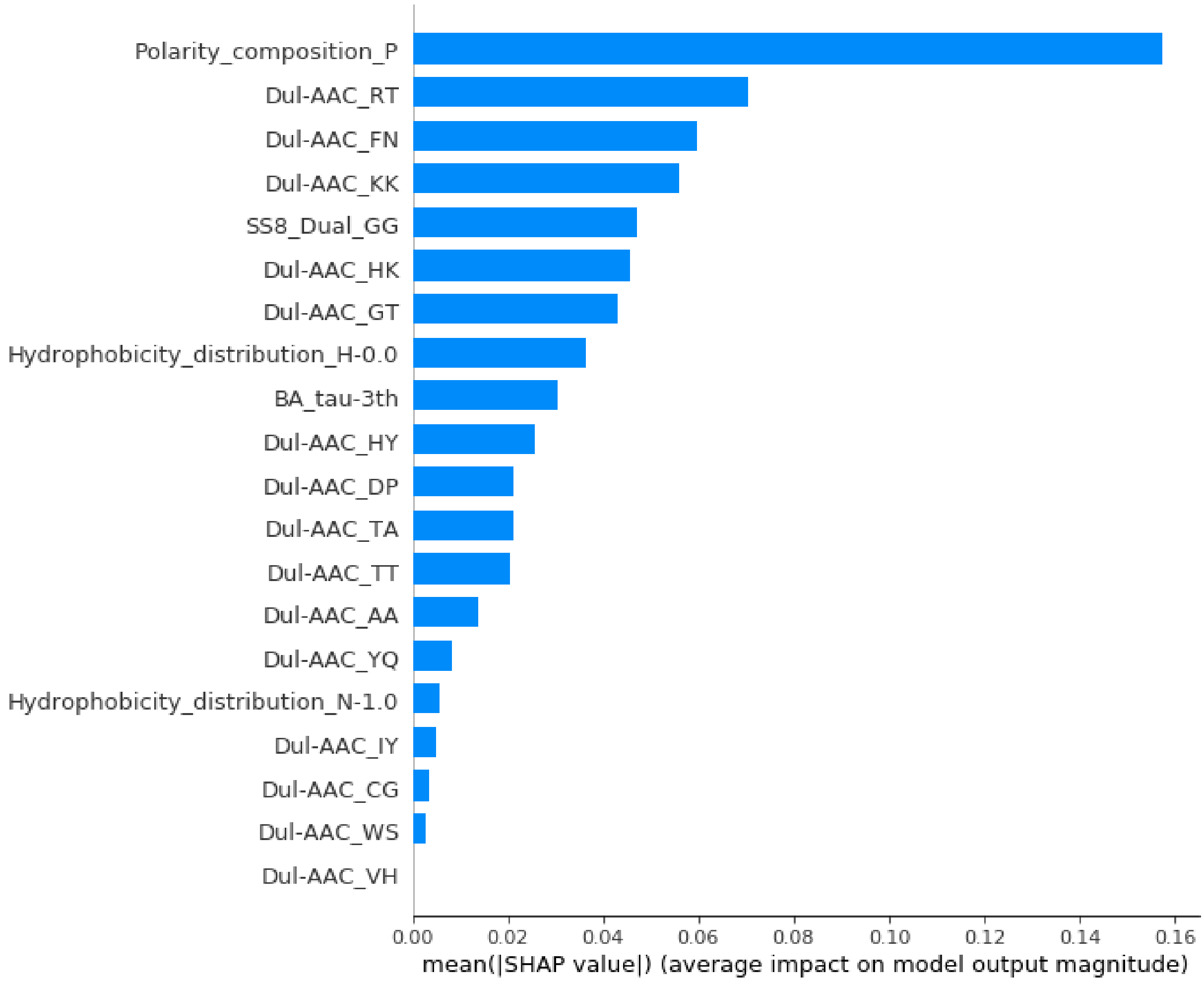

Figure S4: SHAP plot based on 5-fold cross-validation. We calculated the shap_values for every model and its corresponding 17 validation RBPs, merged 5 shap_values into one explainer, and visualized this explainer using SHAP bar plot.

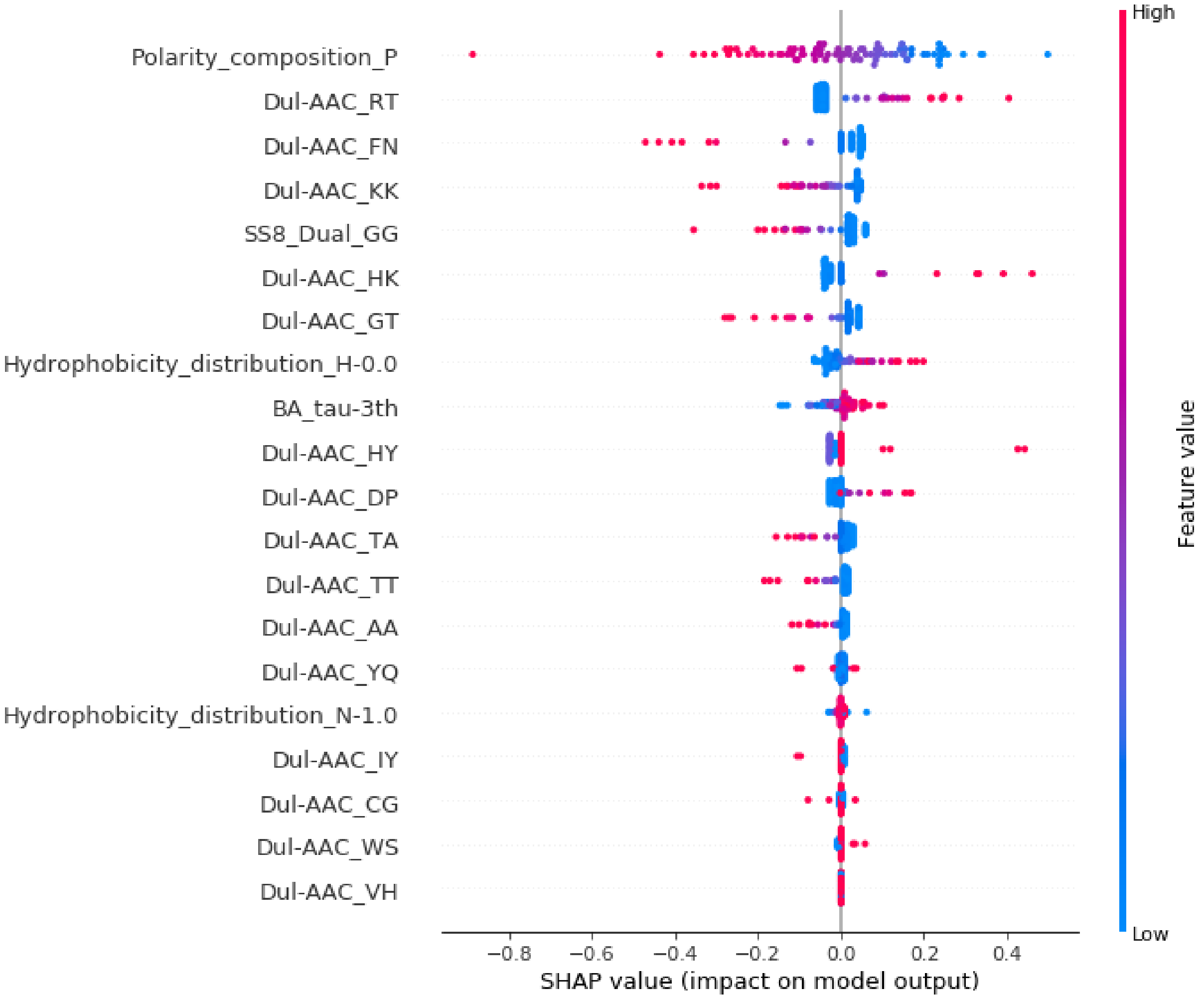

Figure S5: SHAP plot based on 5-fold cross-validation. We calculated the shap_values for every model and its corresponding 17 validation RBPs, merged 5 shap_values into one explainer, and visualized this explainer using SHAP beeswarm plot
